# Angiotensin 1–7 and the Non-Peptide MAS-R Agonist AVE0991 Inhibit Breast Cancer Cell Migration and Invasion

**DOI:** 10.3390/biomedicines13030567

**Published:** 2025-02-24

**Authors:** Mariam M. Alfoudiry, Maitham A. Khajah

**Affiliations:** Department of Pharmacology and Therapeutics, College of Pharmacy, Kuwait University, P.O. Box 24923, Safat 13110, Kuwait; mariam.abdulnabi@grad.ku.edu.kw

**Keywords:** breast cancer, EMT, endocrine resistance, Ang II, Ang 1–7, AVE0991, motility, invasion

## Abstract

**Background:** Endocrine resistance in breast cancer is associated with the epithelial-to-mesenchymal transition (EMT), resulting in enhanced cell proliferation, motility, and invasion and leading to a poor prognosis. There are few studies regarding the role of Angiotensin II (Ang II) and Angiotensin 1–7 (Ang 1–7) in relation to breast cancer, with contradictory outcomes. This study aims to investigate the expression of Ang 1–7 and MAS-R and evaluate the effects of Ang II, Ang 1–7, and the MAS-R agonist AVE0991 on EMT induction and reversal. **Methods:** The effects of Ang II and Ang 1–7 on normal and breast cancer cell lines were determined using various techniques for cell proliferation (MTT), motility (scratch assay), and invasion (Cultrex assay). Also, the expression/localization profiles of Ang 1–7 and its receptor (MAS-R), as well as various EMT markers, were determined using immunofluorescence, western blot, and ELISA. **Results:** Ang II significantly decreased the motility of the tested cell lines; however, it did not have a significant effect on their proliferation or invasion. The expression profiles of the tested EMT markers were not affected by Ang II treatment. The expression levels of Ang 1–7 and MAS-R were significantly higher in the normal breast epithelial cells and estrogen receptor ER compared to the ER+ breast cancer cells. Treatment with Ang 1–7 or the non-peptide MAS-R agonist AVE0991 significantly reduced the migration and invasion of the tested cell lines without modulating the tested EMT markers. Compared to Ang 1–7, AVE0991 exhibited a more prominent dose-dependent inhibitory effect on the proliferation, motility, and invasion of the ER− breast cancer cells. **Conclusions:** Ang 1–7 and AVE0991 play a promising therapeutic role in breast cancer, in part by reducing cell motility and invasion.

## 1. Introduction

Breast cancer is the second most frequently diagnosed form of cancer worldwide, with an estimated incidence of one in every eight women diagnosed with this disease in 2022. According to the latest data from the World Health Organization (WHO), there were 2.3 million women diagnosed with breast cancer and 670,000 deaths globally in 2022. In 2018, breast cancer was the most diagnosed form of cancer among females and the leading cause of malignancy across all ages and genders in the Arabian Gulf region. Kuwait had the highest rates of breast cancer among females in the Arabian Gulf countries [1], and according to the Global Cancer Observatory of the World Health Organization, in 2020, approximately 2002 Kuwaiti females were diagnosed with various types of cancer, of which 791 (39.5%) were breast cancer cases. Most cases of breast cancer are classified as ER+, and estrogen plays a primary role in their growth and proliferation. Patients with ER+ breast cancer are mainly treated with anti-estrogens. However, approximately 30% of breast cancer cases do not express ER (termed ER−, endocrine-resistant form) and are often associated with resistance to endocrine-based therapies, advanced disease stage, and poor prognosis [2,3]. Furthermore, endocrine resistance is also associated with the transformation of cells from an epithelial to a mesenchymal phenotype (termed epithelial-to-mesenchymal transition; EMT). EMT enhances cell proliferation, motility, and invasive capabilities. Thus, EMT is considered a critical step in cancer progression from the pre-invasive to invasive stages due to the ability of cancer cells to leave the primary cancer site and migrate to distant sites, which is associated with a poor prognosis [4,5,6].

Many challenges are encountered during the treatment course, including resistance, serious side effects, and disease recurrence. Therefore, there is a need to better understand disease pathogenesis and to find safer and more potent treatment options. One promising area of research in various disease conditions, such as cardiovascular, renal, and inflammatory disorders, is the polypeptide angiotensin 1–7 (Ang 1–7), which is part of the Renin–Angiotensin–Aldosterone System (RAAS). RAAS is a hormonal system that controls blood pressure and maintains fluid and electrolyte homeostasis. The pathway is activated by angiotensinogen (Agt), which is produced by the liver and is later cleaved by the renin enzyme to form angiotensin I (a weak vasoconstrictor). Angiotensin I is further converted to angiotensin II (Ang II) by an enzyme produced in the lung called the angiotensin-converting enzyme (ACE). Ang II induces various downstream effects when it binds to its G protein-coupled receptors: Ang II receptor type 1 (AT1) and Ang II receptor type II (AT2). Furthermore, Ang II is converted to Ang 1–7 by the action of the ACE. Alternatively, Ang 1–7 can be produced when Ang I is cleaved by the same ACE into Ang 1–9, which is further cleaved to form Ang 1–7 [7,8]. Several reports have suggested that genetic variants in RAAS genes are associated with an increased risk of developing various forms of cancers, such as basal cell carcinoma [9], breast cancer [10], and endometrial cancer [11], and they are associated with poorer prognosis.

Ang II has been shown to play a role in cancer cell proliferation and acts as a mitogen [12]. Some reports have suggested that Ang II promotes tumor growth and the EMT process by targeting the AKT, ERK, and TGFβ/Smad signaling pathways, leading to the subsequent inhibition of E-cadherin in a Snail1-Smad3/4-dependent manner [8,13]. Due to the critical role of ACE in the synthesis of Ang II, it has been shown that reduced ACE levels or the use of ACE inhibitors lowers the risk of breast cancer by 50% [14]. Interestingly, there is a single report suggesting EMT induction (decreased E-cadherin and increased N-cadherin expression) in response to Ang II treatment (0.1 µM, 72 h) using murine normal mammary epithelial cells, NMuMG [13]. This finding was not confirmed by other studies and did not test this effect on normal breast epithelial cell lines of human origin. We aimed to determine the effect of daily treatment with Ang II on MCF10A (normal human breast epithelial cells) to confirm the previous finding in terms of its impact on cell morphology, proliferation, motility, and the expression of selected EMT markers.

As part of the RAAS pathway, the heptapeptide Ang 1–7 is produced mainly as a result of the action of ACE2 on Ang II. Ang 1–7 is considered a mediator of the RAAS pathway and mainly works by counteracting the actions of Ang II, as observed in different pathophysiological settings, including cardiovascular, renal, and immunological disorders. Some reports have shown that treatment with ACE inhibitors results in increased circulating levels of Ang 1–7, which further supports the concept that Ang 1–7 opposes the actions of Ang II. Currently, there are few studies regarding the effects of Ang II and Ang 1–7 in breast cancer cells, with contradictory outcomes. Ang II treatment modulated the expression profile of some EMT markers in different ways, depending on the nature of the treatment, either in vitro or in vivo [15], and the effect of Ang II or Ang 1–7 on normal breast epithelial cells of human origin (e.g., MCF10A) has not been tested previously. In regard to breast cancer cell lines, some studies have shown enhanced MDA-MB-231 cell proliferation in response to Ang II treatment [8,12,14,16,17], while this was not observed in other studies [18]. Also, Ang II reduced MCF-7 [19] while increasing MDA-MB-231 cell invasion [13]. Regarding the effect of Ang 1–7, it inhibited MCF-7 [20,21,22], while it did not affect MDA-MB-231 cell proliferation [13]. To the best of our knowledge, the effect of the non-peptide MAS-R agonist (AVE0991) has not been previously assessed in breast cancer cells, which have several advantages over Ang 1–7 in terms of a lower degradation rate and better stability. Therefore, based on these limiting and contradictory findings, we aimed in this study to determine the role of Ang II, Ang 1–7, and AVE0991 in various normal, ER+, and ER− breast cancer cells regarding their morphology, proliferation, motility, and invasion, as well as the expression profile of selected EMT markers.

## 2. Materials and Methods

### 2.1. Cell Lines and Culture Conditions

The normal breast epithelial cell line MCF10A was obtained from Dr. E. Saunderson and Dr. Jenny Gomm at St. Bartholomew’s Hospital, London. The de novo endocrine-resistant ER− MDA-MB-231 breast cancer cells were obtained from the American Type Culture Collection (ATCC, VA, USA). The acquired endocrine-resistant ER− breast cancer cell line, pII, was established in our laboratory by transfecting the parental MCF-7 with an shRNA plasmid targeting the ER sequence, as previously described [3]. The YS1.2 (ER+) cell line was transfected by an shRNA plasmid but failed to downregulate ER and, therefore, was used as a control for the pII cells [23].

The MDA-MB-231, pII, and YS.2 cell lines were cultured in a 37 °C incubator at 95% humidity and 5% CO_2_ as monolayers in Dulbecco’s Modified Eagle Medium (DMEM), which contained phenol red and was supplemented with 10% fetal bovine serum (FBS), 600 µg/mL L-glutamine, 100 U/mL penicillin, 100 µg/mL streptomycin, and 6 mL/500 mL 100 × non-essential amino acids (all from Invitrogen, CA, USA). MCF10A were cultured in DMEM F12 (Cytiva, Cat# SH30023.01, Marlborough, MA, USA) supplemented with 5% horse serum, 1× Pen/Strep, 20 ng/mL mouse epidermal growth factor (EGF), 0.5 µg/mL hydrocortisone, 100 ng/mL cholera toxin, and 10 µg/mL insulin.

The abovementioned cell lines are routinley used in breast cancer research and represent endocrine-responsive phenotypes (MCF7 and YS1.2), which are less motile and invasive, or endocrine-resistant phenotypes, either de novo (MDA-MB-231) or acquired (pII), which are highly motile and invasive.

### 2.2. Treatment Regimens

Ang II and Ang 1–7 (Sigma-Aldrich) were prepared by dissolving the powder in filtered water to make stock solutions of 10 mM, which were stored at −20 °C. Prior to use, the stock solutions were further diluted with DMEM to achieve the required concentrations (0.1, 1, and 10 µM). AVE0991 was prepared by dissolving the powder in filtered dimethyl sulfoxide (DMSO, Sigma™) to make a stock solution of 1 mM, which was also stored at −20 °C. Prior to use, the stock was further diluted with DMEM to achieve the required concentrations (0.1, 1, and 10 µM). The tested cell lines were treated with Ang II, Ang 1–7, or AVE0991 for various time points (24–96 h). The doses and time points were chosen based on previous reports [9,10,15,18].

### 2.3. Ang 1–7 Levels

Cells (1 × 10^6^) were cultured in a 6-well plate with DMEM to reach 70–80% confluency and then washed with PBS and dissociated using TrypE. The cell suspension was collected into a centrifuge tube and centrifuged for 5 min at 3000 rpm. The supernatant was discarded, and the pellet was resuspended in 5 mL of PBS for washing and centrifuged for 5 min at 3000 rpm. The supernatant was then discarded, and the washing was repeated three times. Cells were lysed in an ultrasonic cell disrupter for 15 min (on ice). Finally, the cells were centrifuged for 10 min at 3000 rpm, and the supernatant was collected into a new 2 mL tube to carry out the assay.

The ready-made Ang 1–7 ELISA kit (MyBioSource, San Diego, CA, USA Cat # MBS2515945) was used. First, the standard working solution was prepared by adding 1 mL of the Reference Standard and Sample Diluent to the powder-form standard vial and was incubated for 10 min at room temperature. Seven serial dilutions were prepared by adding 500 µL of the Reference Standard and Sample Diluent. After that, 500 µL of the working solution was added to the first tube (with the highest concentration). Then, serial dilutions were made in the other tubes, with the last tube containing only the Reference Standard and Sample Diluent and no working solution (blank). The 100× Concentrated Biotinylated Dilution Ab was diluted with Biotinylated Dilution Ab Diluent to a 1× working solution. Similarly, the 100× Concentrated HRP Conjugate was diluted to a 1× working solution with the Concentrated HRP Conjugate Diluent. After this, 50 µL of the standard concentrations were loaded into triplicate wells, starting with 1000 pg/mL. Next, 50 µL of cell lysates were loaded into triplicate wells. The 1× Ab working solution was then immediately added, and the wells were sealed and incubated for 45 min at 37 °C. After that, the solution was removed, and the wells were washed three times with the washing buffer provided in the kit. After that, 100 µL of the 1× HRP Conjugate working solution was added, and the plate was sealed and incubated for 30 min at room temperature. Next, the solution was removed, and the wells were washed five times with the washing buffer. In the dark, 90 µL of the provided Substrate Reagent was added to each well, sealed, and incubated for 15 min. Finally, 50 µL of the provided Stop Solution was added to each well. The plate was read using the MULTISCAN SPECTRUM spectrophotometer (Thermo Scientific) at 450 nm.

### 2.4. Effect of Treatment with Ang II, Ang 1–7, or AVE0991 on Cell Morphology

Cells (1 × 10^6^) were treated with various concentrations of Ang II, Ang 1–7, AVE0991, or vehicle (control) for 24–72 h, and images (40× magnification) were taken daily to monitor any changes in cell morphology using the Nikon Eclipse Ti bright light microscope.

The images were taken and analyzed using the NIS Elements software (https://www.microscope.healthcare.nikon.com/products/software/nis-elements, accessed on 18 February 2025).

### 2.5. Immunofluorescence Analysis

Approximately 5000 cells were seeded into an 8-well chambered slide containing 300 µL of DMEM per well, and the cells were left to settle for 48 h. After that, the DMEM was discarded, and the cells were fixed with 200 µL/well of 3.7% formaldehyde (prepared in PBS) for 10 min with gentle agitation at room temperature and then washed with ice-cold PBS. To block the non-specific binding sites, the cells were treated with 5% Bovine Serum Albumin (BSA, Sigma, prepared in 1× PBS) and incubated for 1 h at room temperature. After that, the BSA was discarded, and 200 µL/well of the primary antibodies, including MAS receptor (Santa Cruz, 1:100 dilution), E-cadherin, and vimentin (Cell Signalling, 1:100 dilution), were added and incubated overnight at 4 °C. The primary antibody solution was removed, and the cells were washed with ice-cold PBS, followed by the addition of the secondary antibodies and incubation for 2 h at room temperature in the dark. The secondary antibody solution was removed, and the cells were washed three times with ice-cold PBS. After that, the Alexa Fluor™ Plus 555 phalloidin (Cat #A30106, Invitrogen, Carlsbad, CA, USA) was prepared at a 1:100 dilution in PBS, and 200 µL was added to each well and incubated for 10 min at room temperature in the dark. The phalloidin was then discarded, and the cells were washed three times with ice-cold PBS. A drop of 4′,6-diamidino-2-phenylindole (DAPI, Cat #D1306, 1 µg/mL) was added and incubated for 5 min at room temperature in the dark. The DAPI was discarded, and the slides were washed three times with ice-cold PBS. The chamber walls were removed using the black slide separator provided with the chamber slides. Finally, a coverslip was mounted on the slides using a drop of mounting media before visualizing the cells under the LSM 800 Meta confocal microscope at 40 and 60× [24].

### 2.6. Cell Proliferation (MTT) Assay

Cells were seeded into 96-well plates at 23,000 cells/well. On the next day, the medium was aspirated, and cell quantity was determined immediately (0 h, seeding day), as well as at 24 h and 96 h of treatment with Ang II, Ang 1–7, AVE0991, or vehicle (control) by removing the medium and adding 80 µL of DMEM and 20 µL of MTT reagent (5 mg/mL). The plates were incubated for 3–4 h at 37 °C. After that, the MTT reagent was aspirated, and 200 µL of DMSO was added to dissolve the blue formazan crystals. Finally, the plates were scanned using a MULTISCAN SPECTRUM spectrophotometer at 570 and 650 nm, after 5 min of shaking, to measure cell proliferation [24,25].

### 2.7. Cell Motility (Scratch) Assay

Cells were cultured in 24-well plates to reach 80–90% confluency. Using a 100 µL pipette tip, a scratch was made along the middle line of the cell monolayer. After that, the media and cell debris were aspirated and replaced with fresh media containing vehicle (control), Ang II, Ang 1–7, or AVE0991. An initial image was taken immediately (0 h) using an inverted microscope, and then the plate was incubated in a 37 °C incubator at 5% CO_2_. After overnight incubation, the scratched area was imaged again (24 h). The width of the scratched area was measured at three places using a scale bar, and the width of the scratch at 24 h was calculated as a percentage of the width of the 0 h scratch [25,26].

### 2.8. Cell Invasion Assay

Cells (1 × 10^6^) were serum-starved (using serum-free DMEM containing 0.5% FBS) overnight in a 37 °C incubator at 5% CO_2_. After that, the cells were washed with PBS, harvested using trypE, transferred to a 15 mL tube, and centrifuged at 3000 rpm for 10 min to obtain the pellet. The cells were then counted and resuspended at 1 × 10^6^ cells/mL in a serum-free medium containing vehicle, Ang II, Ang 1–7, or AVE0991 (10 µM) and added to the top chamber (insert part). The bottom chambers were filled with 500 µL of complete DMEM containing 25% FBS (used as a chemoattractant). The plate was incubated for 24–48 h. After that, the media from the bottom chamber were transferred to a tube and centrifuged at 1500 rpm for 10 min. The supernatant was discarded, the pellet was resuspended in 1 mL of medium, and the cells were counted using a hemocytometer.

### 2.9. Membrane Ruffling Assay

Cells were seeded into well slides at 4–5 × 10^5^ cells/well until reaching 90% confluency and then gently washed three times with ice-cold PBS. Next, a 1.8% agarose solution was prepared by dissolving 0.18 g of agarose in 10 mL of PBS. Once the agarose solution cooled to about 40 °C, 10 µg/mL of EGF (Sigma-Aldrich, St. Louis, MO, USA, E4127, used as a chemoattractant) was added, and the solution was poured toward the edge of the tilted well slide to create a concentration gradient. After that, 2 mL of DMEM (containing vehicle, Ang 1–7, Ang II, or AVE0991) was added to the wells and incubated at 37 °C in a 5% CO_2_ incubator. After 24 h, the DMEM was removed, and the wells were washed three times with ice-cold PBS. Then, the cells were fixed with 3.7% formaldehyde for 10 min at room temperature. Finally, the cells were blocked with BSA and stained as previously described in the immunofluorescence methodology.

### 2.10. Western Blot

Cells were cultured in 6-well plates with DMEM until reaching 80–90% confluency. After that, the media was removed, and the cell monolayer was washed with ice-cold PBS. A homogenization buffer was prepared using a ready-made lysis buffer (Thermo Scientific™, Emeryville, CA, USA) supplemented with protease inhibitors; for each 1 mL of lysis buffer, 1 µL of aprotinin, 1 µL of leupeptin, and 10 µL of phenylmethylsulfonyl fluoride (PMSF) were added (all from Sigma™). After that, 300 µL of the homogenization buffer was added to each well, and the cells were harvested by scraping them off and transferring them to a new Eppendorf tube, which was then centrifuged at 1500 rpm for 10 min. Then, 300 µL of the supernatant was collected in a 2 mL Eppendorf tube and mixed with an equal volume of loading dye that was prepared by adding 50 µL of β-mercaptoethanol to 950 µL of the 2× Laemmli sample buffer (Bio-rad™, Hercules, CA, USA). The samples were boiled for 10 min in a hot water bath and then cooled for another 10 min at room temperature.

Protein concentration was determined using the Bradford assay, with BSA (Sigma™) used as the standard. For the assay, 10 µL of the protein sample was mixed with 99 µL of Coomassie Brilliant Blue dye, and 100 µL of the mixture was pipetted into each well of a 96-well plate. The plate was then read on the ELISA plate reader at 595 nm. Unknown protein concentrations were then determined by plotting a standard curve using the Microsoft Excel program.

Ten percent SDS-polyacrylamide running and stacking gels were prepared, and the protein samples were loaded according to the Bradford assay-determined concentrations. Next, the gel was electrophoresed at 95 V for 10 min, after which the voltage was increased to 135 V and run for another 45 min. After that, the protein bands were transferred to a nitrocellulose membrane and blocked with 5% BSA for 1 h. Primary antibodies (prepared in 5% BSA), β-Actin (loading control) and E-cadherin (1:1000 dilution, Cell Signaling™, Danvers, MA, USA), were added to the blocked membranes and incubated overnight at 4 °C. The membranes were then washed and incubated with an anti-HRP-conjugated secondary antibody (1:1000 dilution for 2 h). Then, the Amersham™ Super Signal ECL™ detection reagent was added to the membrane, and the protein bands were visualized using the ChemiDoc™ MP imaging system [27].

### 2.11. Statistical Analysis

Differences between the mean values of the experimental groups and the controls were determined using one-way ANOVA. The normality of the data was tested using the Shapiro–Wilk and Kolmogorov–Smirnov tests. Using GraphPad Instant software (version 3), statistical significance was considered at *p*-values ˂ 0.05. GraphPad Prism 6 was used to plot the graphs.

## 3. Results

The treatment regimens with Ang II, Ang 1–7, and AVE0991 were performed daily for several time points (3, 5, and 10 days). It should be noted that daily treatments beyond day 4 resulted in cell death in all cell lines; therefore, those time points were excluded. Furthermore, daily treatments with AVE0991 resulted in cell death; thus, the results are presented for a single treatment with AVE0991 performed on day 0.

### 3.1. Effect of Ang II Treatment on the Morphology of MCF10A Cells

**Figure 1:**
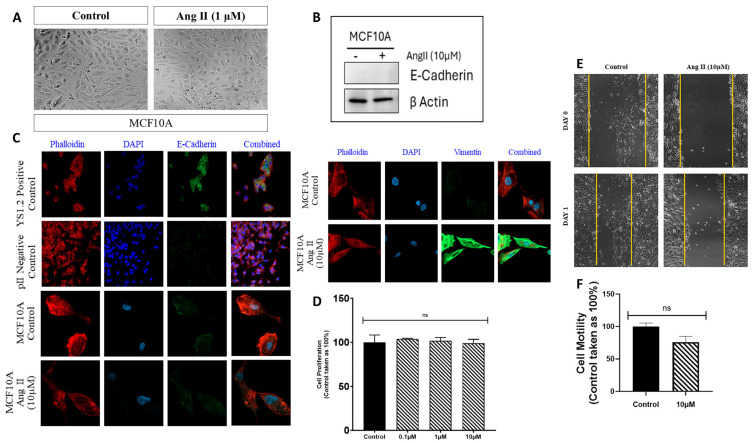
Effect of Ang II treatment on MCF10A cells. The effect of Ang II treatment (1 µM, 72 h) on (**A**) cell morphology (20× magnification) and the expression profile of E-cadherin and vimentin were determined using western blot (**B**) and immunofluorescence analysis (40× magnification, (**C**)). The effect of Ang II treatment (96 h) on cell proliferation was determined in response to vehicle (control, solid bar) or various concentrations of Ang II (hatched bars). Histobars represent means ± SEM of at least 3 independent experiments (n = 3–9 per group, ns: not significant, (panel (**D**))). The degree of cell motility in response to vehicle (control, solid bar) or Ang II (10 µM for 24 h, hatched bar) treatment was determined. Histobars represent means ± SEM of at least 3 independent determinations (n = 3 per group, ns: not significant, (panels (**E**,**F**)), 40× magnification).

First, MCF10A cells were treated with various concentrations (0.1, 1, and 10 µM) of Ang II for 24–72 h, and cell morphology was monitored by capturing an image every 24 h using a light microscope. It was evident that there were no changes in cell morphology in response to Ang II treatment compared to the control (vehicle-treated) cells across all concentrations and time points tested (Figure 1A).

### 3.2. Effect of Ang II Treatment on the EMT Markers of MCF10A Cells

Western blot analysis was performed to determine the effect of Ang II treatment on the expression profile of selected EMT markers. There was no expression of the epithelial marker E-cadherin, either in UT (control) or in response to Ang II treatment (Figure 1B). Then, immunofluorescence analysis was performed to determine the expression profile of E-cadherin, as well as the mesenchymal marker vimentin. E-cadherin was not expressed in MCF10A cells, either in UT (control) or in response to Ang II treatment, confirming the western blotting data. It should be noted that the ER+ breast cancer cell line YS1.2 was used as a positive control, which showed E-cadherin expression using the IF technique (Figure 1C). Vimentin was not expressed in UT cells, but its expression was increased in response to Ang II treatment (Figure 1C).

### 3.3. Effect of Ang II Treatment on MCF10A Cell Proliferation and Motility

Next, we wanted to determine the effect of Ang II treatment on cell proliferation using the MTT assay. Cells were treated with vehicle (control) or various concentrations (0.1, 1, and 10 µM) of Ang II for 96 h. Ang II treatment did not modulate cell proliferation compared to the control group (Figure 1D). The effect of Ang II treatment on cell motility was assessed using the scratch assay. It should be noted that MCF10A cells are not motile in the absence of any chemoattractant; therefore, DMEM containing 10 µg/mL of EGF (used as a chemoattractant) was used to induce MCF10A motility. Ang II treatment (10 µM for 24 h) did not modulate cell motility compared to vehicle-treated cells (control, Figure 1E,F).

### 3.4. Effect of Ang II Treatment on the Morphology of ER− and ER+ Breast Cancer Cells

**Figure 2:**
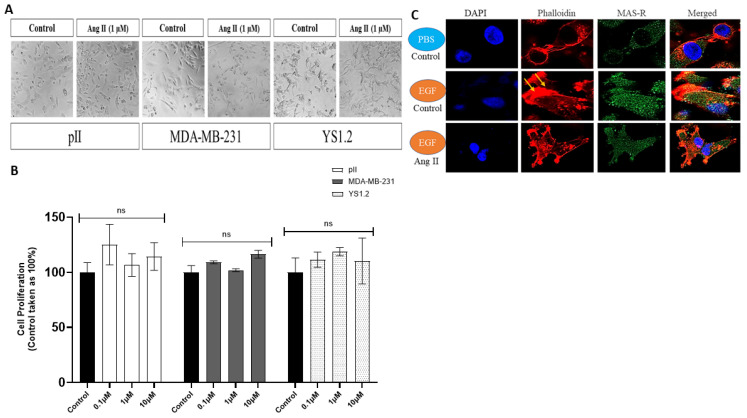
Effect of Ang II treatment on ER+ and ER− breast cancer cells. The effect of Ang II treatment (1 µM, 72 h) was determined on cell morphology (20× magnification, (panel (**A**))). The effect of Ang II treatment (96 h) on cell proliferation was determined in response to vehicle (control, solid bars) or various concentrations of Ang II. Histobars represent means ± SEM of at least 3 independent experiments (n = 9–21 for control groups, and n = 3–9 per various treatment groups, ns: not significant, (panel (**B**))). (Panel (**C**)) shows immunofluorescence analysis of pII cells in response to Ang II pre-treatment (10 µM for 24 h) and a concentration gradient of EGF (10 µg/mL) or PBS (used as negative control). Cells were stained with phalloidin (red, for actin cytoskeleton), MAS-R (green), and DAPI (blue, for the nucleus). Membrane ruffling was evident (yellow arrows) in response to a concentration gradient of EGF (63× magnification) (ns = not significant).

Next, we wanted to determine the effect of Ang II treatment on breast cancer cells. ER− (pII and MDA-MB-231) and ER+ (YS1.2) breast cancer cell lines were treated with various concentrations (0.1, 1, and 10 µM) of Ang II for 72 h, and cell morphology was monitored every 24 h under a light microscope. Ang II treatment did not modulate cell morphology in the tested cell lines compared to the vehicle-treated group (control, Figure 2A).

### 3.5. Effect of Ang II Treatment on the Proliferation of ER− and ER+ Breast Cancer Cell Lines

ER− (pII and MDA-MB-231) and ER+ (YS1.2) breast cancer cells were treated with various concentrations (0.1, 1, and 10 µM) of Ang II for 96 h, and cell proliferation was determined using the MTT assay. Ang II treatment did not modulate proliferation in any of the tested cell lines (Figure 2B).

### 3.6. Effect of Ang II Treatment on Membrane Ruffling in pII Cells

We next determined if Ang II treatment affects actin cytoskeleton rearrangements in pII cells in response to a concentration gradient of epidermal growth factor (EGF, used as a chemoattractant, 10 µg/mL). Actin cytoskeleton rearrangement plays an essential role in cell motility potential. Cells were either pre-treated with vehicle (PBS, negative control) or Ang II (10 µM for 24 h) and then seeded into a 6-well petri dish with EGF placed in an agarose spot on the side of the dish (to establish a concentration gradient). Membrane ruffling (yellow arrows) was evident in the cell direction facing the EGF but not in the PBS gradient. Ang II treatment reduced the degree of membrane ruffling in response to the EGF gradient. The MAS-R (the receptor for Ang 1–7, used herein as a negative control) expression pattern was not affected by Ang II pre-treatment or in response to the EGF concentration gradient (Figure 2C).

### 3.7. Effect of Ang II Treatment on the Migration of ER− and ER+ Breast Cancer Cell Lines

**Figure 3:**
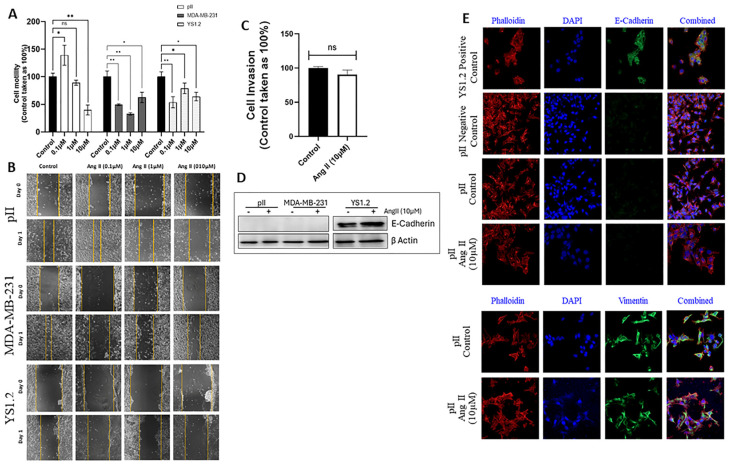
Effect of Ang II treatment on breast cancer cell motility and invasion and the expression of selected EMT markers. The degree of pII, MDA-MB-231, and YS1.2 cell motility in response to vehicle (control, solid bar) or various concentrations of Ang II (colored bars) treatments was determined. Histobars represent means ± SEM of at least 3 independent determinations (n = 3 per group, ns: not significant). Asterisks denote the significant differences compared to control: * *p* ˂ 0.05 and ** *p* ˂ 0.001 (panels (**A**,**B**), 20× magnification). pII cell invasion was determined in response to treatment with vehicle (control, solid bar) or with Ang II (10 µM, white bar) (n = 3 per group, ns: not significant, (panel (**C**))). The effect of Ang II treatment (10 µM, 24 h) on the expression profile of E-cadherin and vimentin was determined using western blot and (**D**,**E**) immunofluorescence analysis (40× magnification) (ns= not significant).

The effect of Ang II treatment (0.1, 1, and 10 µM, 72 h) on cell migration was determined using the scratch assay. pII cells demonstrated increased migration at the 0.1 µM Ang II concentration; however, there was a significant reduction in pII cell migration at the 10 µM concentration of Ang II [which also reduced the degree of membrane ruffling (Figure 2C)]. Ang II significantly reduced MDA-MD-231 and YS1.2 cell motility at all tested concentrations (Figure 3A,B).

### 3.8. Effect of Ang II Treatment on pII Cell Invasion

The effect of Ang II treatment (10 µM) on pII cell invasion was determined using a cultrex assay. Ang II did not modulate pII cell invasion compared to vehicle-treated cells (control, Figure 3C).

### 3.9. Effect of Ang II Treatment on the EMT Markers in ER+ and ER− Breast Cancer Cells

The epithelial marker E-cadherin is only expressed in the ER+ breast cancer cells, while the mesenchymal marker vimentin is only expressed in the ER− breast cancer cells. The levels of these markers were tested using western blot and immunofluorescence techniques. It was evident that Ang II treatment did not modulate the expression of these markers in the tested cell lines (Figure 3D,E).

### 3.10. Ang 1–7 Levels in Breast Cancer and Normal Breast Epithelial Cell Lines

**Figure 4:**
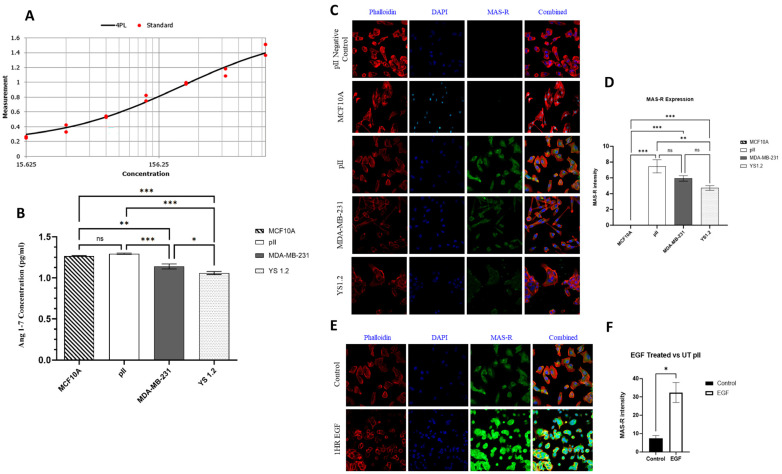
The expression profile of Ang 1–7 and MAS-R in the tested cell lines. The endogenous Ang 1–7 expression level in the tested cell lines was determined using ELISA technique. (Panel (**A**)) shows the standard curve calculated with a four-parametric logistic assay (4PL). (Panel (**B**)) shows Ang 1–7 concentration (pg/mL). Histobars represent means ± SEM of 3 independent determinations (n = 3 per cell line, ns: not significant). Asterisks denote significant differences: * *p* ˂ 0.05, ** *p* ˂ 0.001, and *** *p* ˂ 0.0001. (Panels (**C**,**D**)) show MAS-R expression pattern/level in the tested cell lines using immunofluorescence and semi-quantitative measurements with Image J software (version 2) (40× magnification). (Panels (**E**,**F**)) show MAS-R expression pattern/level in pII cells in response to EGF treatment (100 ng, 1 h, 40× magnification). Histobars represent means ± SEM of 3 independent determinations (n = 3 per cell line, ns: not significant). Asterisks denote significant differences: * *p* ˂ 0.05, ** *p* ˂ 0.001, and *** *p* ˂ 0.0001.

Using the ELISA technique, the endogenous Ang 1–7 expression level was determined in all of the tested cell lines. First, a standard curve was calculated using the four-parametric logistic assay (4PL), and the kit met the manufacturer’s recommended levels (Figure 4A). After that, the experiment was carried out to determine the level of Ang 1–7 in the tested cell lines. As shown in Figure 4B, the expression level of Ang 1–7 in the ER− breast cancer cells (MDA-MB-231 and pII) was significantly higher compared to the ER+ breast cancer cells (YS1.2). The expression level of Ang 1–7 in MCF10A was similar to that in pII and MDA-MB-231 cells (Figure 4B).

### 3.11. MAS-R Expression Level in the Tested Cell Lines

MAS-R (receptor for Ang 1–7) expression levels in MCF10A, ER−, and ER+ breast cancer cell lines were determined using immunofluorescent analysis and semi-quantitative measurements. As shown in Figure 4C,D, the expression level of MAS-R (measured using Image J software, version 2) was not evident in MCF10A cells. In contrast, the expression level of MAS-R was significantly higher in breast cancer cell lines compared to MCF10A, with a greater trend of expression in the ER− cancer cells compared to ER+ cancer cells. The MAS-R expression pattern was predominantly localized in the cytoplasm of all tested cell lines. Next, we determined the effect of EGF (100 ng) stimulation on the expression pattern/level in pII cells. Surprisingly, the expression level of MAS-R significantly increased (by twofold) in response to EGF stimulation (Figure 4E,F).

### 3.12. Effect of Ang 1–7 and AVE0991 Treatment on the Morphologies of the Tested Cell Lines

**Figure 5:**
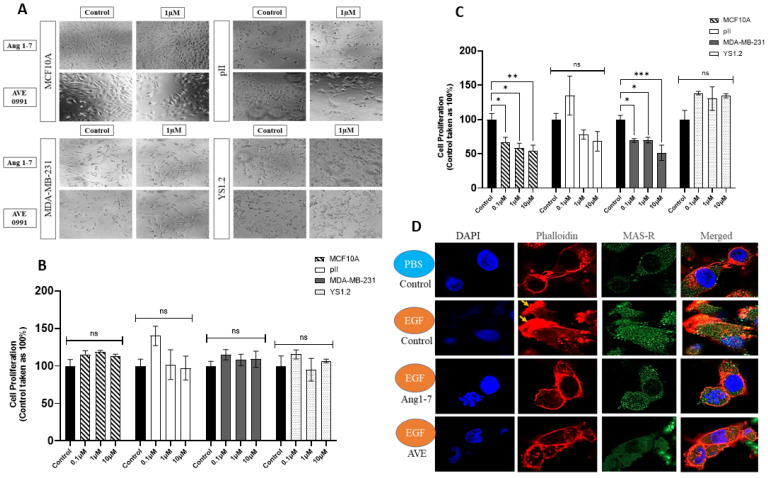
Effect of Ang 1–7 and AVE0991 treatment on the tested cell lines. The effects of Ang 1–7 and AVE0991 treatment (1 µM, 72 h) on cell morphology were determined (20× magnification, (panel (**A**))). The effects of Ang 1–7 (daily treatment regimen, (**B**)) and AVE0991 (single treatment regimen on day 0, (**C**)) treatment on cell proliferation (96 h) were determined. Histobars represent means ± SEM of at least 3 independent determinations (n = 3–9 per group, ns: not significant). Asterisks denote the significant differences compared to control: * *p* ˂ 0.05, ** *p* ˂ 0.001, *** *p* ˂ 0.0001. (Panel (**D**)) shows immunofluorescence analysis of pII cells in response to vehicle, Ang 1–7, or AVE 0991 pre-treatment (10 µM for 24 h) with a source of a concentration gradient of EGF (10 µg/mL) or PBS (negative control, 40× magnification). Cells were stained with phalloidin (red, for actin cytoskeleton), MAS-R (green, used as negative control), and DAPI (blue, for the nucleus). Yellow arrows indicate membrane ruffling (40× magnification) (ns = not significant).

The normal breast epithelial cell line MCF10A, the ER+ breast cancer cell YS1.2, and two ER− breast cancer cells, pII and MDA-MB-231, were treated with various concentrations of Ang 1–7 or AVE0991 (MAS-R agonist) (0.1, 1, 10 µM) for 72 h, and an image was taken every 24 h during the treatment period. Ang 1–7 and AVE0991 treatments did not affect the morphology of any of the tested cell lines, as shown in Figure 5A, which shows treatment with 1 µM for 72 h as an example.

### 3.13. Effect of Ang 1–7 or AVE0991 Treatment on Cell Proliferation

The effects of various concentrations of Ang 1–7 or AVE0991 (0.1, 1, and 10 µM for 72 h) on cell proliferation were determined using the MTT assay. As shown in Figure 5B, Ang 1–7 treatment did not modulate proliferation in any of the tested cell lines. Regarding AVE0991, daily treatment resulted in cell death in all cell lines. Therefore, we used a single treatment regimen on day 0, and cell proliferation was measured on day 4. AVE0991 treatment reduced the proliferation of MCF10A and MDA-MB-231 in a dose-dependent manner. There was a trend of reduced pII cell proliferation (but it did not reach statistical significance), while YS1.2 proliferation was not affected by AVE0991 treatment (Figure 5C).

### 3.14. Effect of Ang 1–7 and AVE0991 Treatment on Membrane Ruffling in pII Cells

pII cells were pre-treated with the vehicle, Ang 1–7, or AVE0991 (10 µM for 24 h) and then seeded into a small dish facing a source of a concentration gradient of EGF (used as a chemoattractant). When pII cells faced a source of PBS (used as a negative control), the cells did not show signs of membrane ruffling (actin cytoskeleton rearrangement or thickening). However, pII cells showed membrane ruffling toward the source of EGF (Figure 5D, yellow arrow), which was abolished with Ang 1–7 or AVE0991 pre-treatment. MAS-R staining (used as a negative control) did not change in response to the EGF gradient under any treatment condition.

### 3.15. Effect of Ang 1–7 or AVE0991 Treatment on Cell Migration

**Figure 6:**
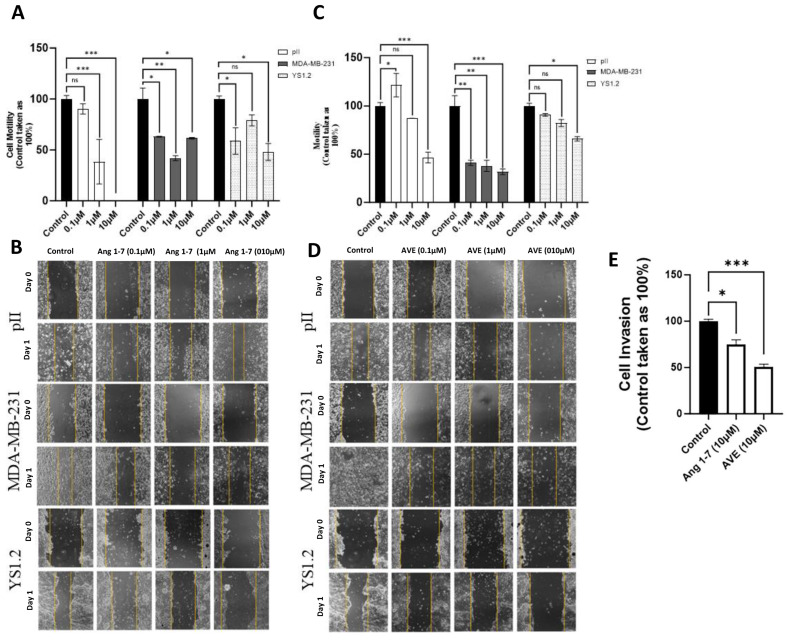
Effect of Ang 1–7 and AVE0991 treatment on breast cancer cell motility and invasion. The degree of cell motility in response to vehicle (control, solid bar) or treatment with Ang 1–7 or AVE 0991 (colored bars) was determined. (Panels (**A**,**B**), 20× magnification) represent cell motility in response to Ang 1–7 treatment. (Panels (**C**,**D**), 40× magnification) represent cell motility in response to AVE0991 treatment. Histobars represent means ± SEM of at least 3 independent determinations (n = 3 per group, ns: not significant). Asterisks denote the significant differences from control: * *p* ˂ 0.05, ** *p* ˂ 0.001, *** *p* ˂ 0.0001. (Panel (**E**)) shows pII cell invasion in response to Ang 1–7 and AVE0991 treatment. Cells were pre-treated with vehicle (control, solid bar), Ang 1–7, or AVE 0991 (10 µM, open bars). Histobars represent means ± SEM of at least 3 independent determinations (n = 3 per group). Asterisks denote the significant differences from control: * *p* ˂ 0.05, *** *p* ˂ 0.0001.

ER− (pII and MDA-MB-231) and ER+ (YS1.2) breast cancer cells were pre-treated with Ang 1–7 or AVE0991 (0.1–10 µM) for 72 h. Then, a scratch was made, and cell motility was monitored after 24 h. Ang 1–7 (Figure 6A,B) and AVE0991 (Figure 6C,D) treatment significantly reduced cell motility in all of the tested cell lines in a dose-dependent manner.

### 3.16. Effect of Ang 1–7 or AVE0991 Treatment on pII Cell Invasion

We previously showed that normal breast epithelial and ER+ breast cancer cells are not invasive. Therefore, we tested the effect of Ang 1–7 or AVE0991 treatment on pII cell invasion using the Cultrex assay. As shown in Figure 6E, Ang 1–7 and AVE0991 treatment significantly decreased pII cell invasion compared to the control.

### 3.17. Effect of Ang 1–7 or AVE0991 Treatment on Selected EMT Markers in Breast Cancer Cells

**Figure 7:**
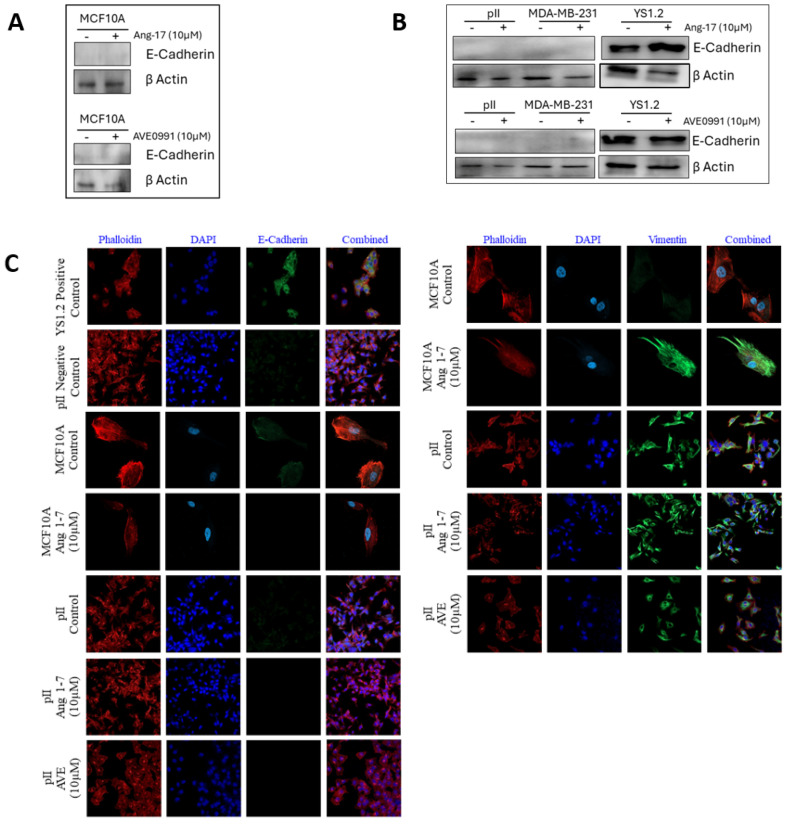
Effect of Ang 1–7 and AVE0991 treatment on the expression profile of selected EMT markers in breast cancer cells. The effect of Ang 1–7 or AVE0991 treatment on the expression profile of E-cadherin and vimentin was determined using western blot (**A**,**B**) and immunofluorescence techniques (**C**, 40× magnification).

Western blot analysis was performed on the MCF10A, pII, MDA-MB-231, and YS 1.2 cells to determine the effect of Ang 1–7 or AVE0991 treatment on the expression profile of E-cadherin. It was previously evident that pII and MDA-MB-231 cells do not express E-cadherin, while YS 1.2 does [28]. Treatments of the different ER+ and ER− breast cancer cell lines and normal breast epithelial cells with Ang 1–7 or AVE0991 did not modulate the E-cadherin expression profile (Figure 7A,B). Next, immunofluorescence analysis was performed to confirm the western blot results of the E-cadherin expression levels, as well as to determine the expression profile of the mesenchymal marker vimentin. E-cadherin was only expressed in the YS 1.2 cells, but not in the MCF10A, pII, or MDA-MB-231 cells, confirming the western blotting data. Moreover, Ang 1–7 and AVE0991 treatments did not modulate the expression profiles of E-cadherin or vimentin (Figure 7C).

## 4. Discussion

Breast cancer is one of the leading causes of mortality worldwide, and de novo or acquired endocrine resistance further complicates the disease prognosis and increases mortality rates [28,29]. Endocrine resistance is often associated with EMT, resulting in enhanced cancer cell proliferation, migration, invasion, and loss of sensitivity to anti-endocrine therapy, leading to a poor prognosis [2,3,5]. The RAAS seems to play a role in cancer progression, with some reports suggesting enhanced cancer cell proliferation and migration in response to Ang II treatment [8,12,13]. In addition, Ang 1–7 is a known counterpart for Ang II, but its role in cancer is still unclear [13,30]. This study investigates the expression of Ang 1–7 and MAS-R and evaluates the effects of Ang II, Ang 1–7, and the MAS-R agonist AVE0991 on EMT induction and reversal.

### 4.1. Effect of Ang II and Ang 1–7 Treatment on MCF10A Cells

#### 4.1.1. Ang II

As previously mentioned, there is no clear evidence regarding the effect of Ang II treatment on normal breast epithelial cells. In a study performed by Piastowska-Ciesielska et al., using normal mammary cells 184A1, they demonstrated enhanced cell migration in response to Ang II treatment (24 h) [31]. In another study by Cambados et al. using murine mammary epithelial cells NMuMG, they demonstrated EMT induction, enhanced cell migration, and invasion in response to Ang II treatment [13]. Another study by De Paepe et al. showed that Ang II treatments on HMec normal mammary epithelial cells did not affect cell proliferation [32]. Therefore, based on these contradicting findings, we aimed to determine the effect of Ang II treatment on the normal breast epithelial cell line of human origin, MCF10A. Cells were treated with various concentrations (0.1, 1, and 10 µM) of Ang II for 24–72 h. We did not observe any change in cell morphology in response to Ang II treatment, which provided indirect evidence that Ang II did not induce EMT (Figure 1A and Figure 2A). During the EMT process, cell polarity and cell–cell contact/adhesion losses are usually associated with decreased expression of the epithelial marker E-cadherin and increased expression of the mesenchymal markers vimentin and N-cadherin. According to a study by Cambados et al., treatment of the non-tumorigenic cell line, NMuMG, with Ang II resulted in reduced E-cadherin and increased N-cadherin expression, suggesting EMT induction [13]. Takiguchi et al. conducted a similar study on the effect of Ang II treatment on EMT-related proteins, and conflicting results were observed depending on the nature of the treatments. In vivo, treatments with Ang II induced EMT by increasing the expression of the EMT-related proteins vimentin, fibronectin, αSMA, and Snail. In contrast, in vitro treatment with Ang II did not alter the expression profile of the same EMT-related proteins [15]. Based on these conflicting studies, we investigated the effects of Ang II on the epithelial and mesenchymal markers in MCF10A cells. Using western blot, we found that the expression profile of E-cadherin in MCF10A cells was undetectable or expressed at very low levels and was not modulated by Ang II treatment (Figure 2A). Immunofluorescence analysis further confirmed this observation (Figure 2B). Interestingly, enhanced vimentin expression was detected in response to Ang II treatment using IF analysis (Figure 1B). Furthermore, Ang II did not modulate MCF10A proliferation and migration (Figure 1D–F). It is interesting that enhanced vimentin expression did not enhance MCF10A motility, which may suggest that these cells did not gain oncologic features in response to Ang II treatment.

#### 4.1.2. Ang 1–7

To the best of our knowledge, no studies have been performed to determine the effect of Ang 1–7 treatment on normal breast epithelial cells. First, the endogenous expression profile of Ang 1–7 in MCF10A cells was measured. It was evident that Ang 1–7 is abundantly expressed in MCF10A cells (Figure 2A,B). Ang 1–7 acts by binding to its G-protein-coupled receptor, MAS-R [13,33,34]; therefore, immunofluorescence analysis was performed to visualize and semi-quantitatively measure MAS-R expression levels in MCF10A cells. Unexpectedly, MAS-R expression levels were negligible or minimal, as shown by immunofluorescence (Figure 4C,D). Other more sensitive techniques must be used to confirm the “low” expression profile of MAS-R in MCF10A cells. Next, the effect of Ang 1–7 treatments on the MCF10A cells was investigated using various concentrations (0.1, 1, and 10 µM) at different time points (24–96 h). Ang 1–7 did not affect the MCF10A cells’ morphology or proliferation (Figure 5A,B). It has been recently demonstrated by Cambados et al. that Ang 1–7 counteracted Ang-II-induced EMT by enhancing E-cadherin and reducing N-cadherin expression [13]. We performed western blot and immunofluorescent analyses and showed that Ang 1–7 enhanced vimentin expression (Figure 7C) without modulating any of the tested effector functions.

### 4.2. Effect of Ang II and Ang 1–7 Treatment on ER− and ER+ Breast Cancer Cells

#### 4.2.1. Ang II

Several studies were performed regarding the effect of Ang II and Ang 1–7 treatment on various breast cancer cells, such as MCF-7 (ER+) and MDA-MB-231 (ER−). Ang II treatment enhanced MCF-7 cell proliferation in a dose- and time-dependent manner [8,12,14,16,17]. However, Ang II treatment did not affect the proliferation of MDA-MB-231 cells [18]. In our study, we showed that Ang II treatment did not alter the proliferation of any of the tested ER+ and ER− cell lines (Figure 2B). These results are in agreement with those of Rodrigues-Ferreira et al., who also did not show a change in cell proliferation in response to Ang II treatment. We next assessed the effect of Ang II on cell migration and invasion. We showed that the rearrangement of the actin cytoskeleton (membrane ruffling, which is essential for cell migration) in response to a concentration gradient of a chemoattractant was reduced in response to Ang II treatment (Figure 2C). Several studies have suggested a significant increase in MDA-MB-231 cell migration in response to Ang II treatment [13,18,35]. In our study, Ang II treatment increased pII migration only at a low dose (0.1 µM), and no modulation in cell migration was observed at higher doses of Ang II. Also, Ang II significantly reduced the migration of MDA-MB-231 and YS1.2 cells (Figure 3A,B). Regarding cell invasion, Puddefoot et al. showed that Ang II reduced MCF-7 cell invasion [19]. In contrast, Ang II treatment increased MDA-MB-231 cell invasion [13]. In our study, we showed that Ang II treatment did not modulate pII cell invasion (Figure 3C). The expression of EMT-related proteins was evaluated using western blot and immunofluorescence. Ang II treatment did not alter the expression of E-cadherin or vimentin in the tested cell lines (Figure 3D,E), suggesting that Ang II did not modulate the EMT process.

#### 4.2.2. Ang 1–7

We first measured endogenous Ang 1–7 levels and showed that the ER− cell lines (pII and MDA-MB-231) expressed significantly higher Ang 1–7 levels than the ER+ cell line, YS1.2 (Figure 4A,B). As mentioned above, Ang 1–7 binds to its G-protein-coupled receptor, MAS-R, and activates it [13,33,34]. Therefore, we proceeded to qualitatively measure the levels of MAS-R in these cell lines using immunofluorescent analysis. MAS-R was highly expressed in the ER− compared to the ER+ breast cancer cells (Figure 4C,D), and EGF stimulation further enhanced (by twofold) its expression in pII cells (Figure 4E,F). Next, the different cell lines were treated with Ang 1–7 at various concentrations and durations, and the cells’ morphology, proliferation, migration, and invasion were assessed. First, no morphological changes in the tested cell lines were observed in response to the Ang 1–7 treatment (Figure 5A). According to several studies conducted on MCF-7 cells, Ang 1–7 inhibited cell proliferation [20,21,22]. However, MDA-MB-231 cells showed no change in proliferation when treated with Ang 1–7 [13]. In our study, Ang 1–7 treatment did not alter proliferation in any of the tested cell lines (Figure 5B). Next, the migration of the different cell lines with Ang 1–7 treatments was determined. As described above, the membrane ruffling of the pII cells in response to the EGF chemoattractant was evaluated. As shown in Figure 5D, Ang 1–7 inhibited membrane ruffling in pII cells toward the source of EGF. Moreover, Ang 1–7 was shown to reduce the migration and invasion rates of the MDA-MB-231 cells [13,36]. Similar results were obtained in our study, in which Ang 1–7 treatments reduced the migration of pII, MDA-MB-231, and YS1.2 cells (Figure 6A–D). Additionally, trans-well invasion assays on pII cells showed reduced invasion in response to Ang 1–7 treatment (Figure 6E). Regarding the effect of Ang 1–7 on the expression profile of EMT markers, Yu et al. showed that Ang 1–7 treatment increased the expression of E-cadherin in MDA-MB-231 cells, resulting in reduced cell invasion [36]. We showed that Ang 1–7 did not affect the expression of E-cadherin or vimentin in the tested cell lines (Figure 7).

### 4.3. Effect of AVE 0991 Treatment on MCF10A, pII, MDA-MB-231, and YS1.2 Cells

One drawback of using Ang 1–7, which is a peptide, is its short half-life and low bioavailability due to rapid enzymatic metabolism by peptidases, including ACE and dipeptidyl peptidase 3, 1, and 9. Also, Ang 1–7 is rapidly degraded in the gastrointestinal tract when administered orally. Therefore, we used AVE0991, which is a non-peptide that acts as a MAS-R agonist and mimics the antihypertensive and antifibrotic effects of Ang 1–7 on various organs [37,38]. To the best of our knowledge, no studies have been performed on the role of AVE0991 in breast cancer cells. First, the cell lines were treated with a single dose of AVE0991 for 72 h, and as shown in Figure 5A, AVE0991 did not alter the morphology of any of the tested cell lines. Next, we measured the proliferation rate of the different cell lines after 96 h of a single treatment with various concentrations of AVE0991 (0.1, 1, 10 µM). It was evident that AVE0991 significantly reduced the proliferation of the MCF10A and MDA-MB-231 cells in a dose-dependent manner. In pII cells, AVE0991 treatment reduced cell proliferation, but this did not reach statistical significance. Furthermore, AVE0991 treatment did not alter YS1.2 cell proliferation (Figure 5B). The effect of AVE0991 treatment on cell migration was also assessed. AVE0991 treatment reduced membrane ruffling in pII cells (Figure 5D) and decreased migration in pII, MDA-MB-231, and YS1.2 cells in a dose-dependent manner (Figure 6C,D). Moreover, AVE0991 significantly decreased pII cell invasion (Figure 6E). Finally, the EMT markers of pII, MDA-MB-231, and YS1.2 cells were measured using western blot and immunofluorescent analyses. Overall, AVE0991 did not modulate the tested cell lines’ E-cadherin or vimentin expression profiles (Figure 7).

Compared to Ang 1–7, AVE0991 had a more prominent dose-dependent inhibitory effect on the proliferation, motility, and invasion of the ER− cells, making it a potentially effective line of treatment despite the lack of change in the expression of the EMT markers, which is seen in both Ang 1–7 and AVE0991 treatments. The difference between the two compounds may be due to the fact that AVE0991 is a non-peptide and is able to resist proteolytic enzymes. AVE0991 may have a stronger binding affinity to MAS-R compared to Ang 1–7, which requires further investigation.

## 5. Conclusions

In conclusion, we showed that Ang II did not modulate various effector functions of different breast cancer cells or normal epithelial cells. Also, Ang II did not induce EMT in normal breast epithelial cells. In addition, Ang 1–7 treatment reduced the migration and invasion of different breast cancer cell lines but did not alter the expression of the tested epithelial or mesenchymal markers. The non-peptide MAS-R agonist AVE0991 showed a significant decrease in the proliferation of MCF10A and ER− breast cancer cells, as well as the migration of the MCF10A, ER−, and ER+ cell lines, but did not alter the expression profile of the tested EMT markers. In the current study, we highlight the effectiveness of Ang 1–7 and AVE0991 as potential therapeutic agents for breast cancer treatment, particularly for the endocrine-resistant form.

A major strength of this study is that it provided insights into the different RAAS components (Ang II and Ang 1–7) and their roles in various effector functions of breast cancer cells. We also highlighted, for the first time, the effect of AVE0991 treatment on the proliferation, migration, and invasion of the different breast cancer types tested. Some limitations and future directions based on this study include the need for more mechanistic insights regarding the role of Ang 1–7 in reducing breast cancer cell motility and invasion. For example, it is necessary to determine the expression/activity profile of several important downstream molecules responsible for cell migration and invasion, such as p38 MAPK, ERK1/2, Akt, and mTOR, as well as other EMT markers not tested in this project (such as fibronectin, cytokeratin, and various MMPs). It should be noted that various signaling molecules downstream of MAS-R are activated and involved in cancer pathogenesis, such as phosphoinositide 3-kinases (PI3k)/protein kinase B (Akt), NADPH oxidase (NOX), AMP-activated protein kinase (AMPK)/forkhead box protein O1 (FoxO1), phospholipase C (PLC), inositol trisphosphate (IP3), p38 mitogen-activated protein kinase (p38 MAPK), and the mammalian target of rapamycin (mTOR), all of which are potential targets for future studies (for review, [39]). Also, the role of Ang 1–7 and AVE0991 in vivo in breast cancer models (either as monotherapy or in combination with existing therapies) needs to be explored. Furthermore, investigating the roles of Ang 1–7 and Ang II in other forms of cancer is warranted to provide a better understanding of their involvement in cancer.

## Data Availability

All data generated or analyzed during this study are included in this published article.
